# ChatGPT in the health sciences: pause and ponder

**DOI:** 10.1186/s12940-023-01014-6

**Published:** 2023-09-14

**Authors:** Ana Paula Razal Dalvi, Rayara Mozer Dias, Washington Leite Junger

**Affiliations:** Department of Epidemiology, Institute of Social Medicine, State University of Rio de Janeiro, Rio de Janeiro, Brazil

**Keywords:** Air Pollution, Artificial Intelligence, Environment and Public Health

The field of Artificial Intelligence (AI) is rapidly evolving in the world and prominently in global health [1]. Since OpenAI launched ChatGPT, scientists have wondered what role Artificial Intelligence (AI) has played in scientific research. There has been much excitement and apprehension as questions for the ChatGPT bot have been posed. This concern has led academics and executives to call for a pause in the development of AI systems with human-competitive intelligence [2]. Soon, AI systems will be available to everyone who uses a web search engine, such as Google or Bing.

We expect scientists to be critical in their pursuit of knowledge. But what about the non-academic-scientific population? It is much easier to seek information by directly asking for it in a chat than undertaking a “laborious” search on Google. For all intents and purposes, today’s chat is tomorrow’s Google. Eventually, instead of having a range of possible information sources on a subject of interest, we will have one single answer and, quite possibly, one that doesn’t include its sources. This is much more straightforward, but also much more dangerous.

Studies demonstrate that promising ChatGPT applications can influence health care education and research shifts. However, considering its possible restrictions, adopting this AI chatbot must be ethical, responsible, and conducted with extreme caution [3]. These made us question: Is the information provided by AI reliable? Or do we have a tool which, in a world of growing anti-science groups, will do more harm than good? In order to address some of these questions, we conducted a chat on ChatGPT using environmental epidemiology as our frame of reference. We asked one broad question, “What is the relationship between air pollution and mortality?“, and a specific one, “What is the relative risk of exposure to air pollution on respiratory mortality in Europe?“.

Our first surprise came when we identified citations in the answer from studies published in important public health journals: The Lancet, The Lancet Planetary Health, and Environmental Health Perspective (EHP). However, since we are scientists and “always” ponder on what we read, we also investigated whether these studies were cited correctly. When we experienced difficulties in locating them, we asked ChatGPT for help and requested more information about the studies it referred to. At this point, we began to be concerned about the answers.

The answer to the first question was that a study published in The Lancet in 2019 provided an estimate that air pollution contributed to 8.8 million premature deaths in 2015, citing a study entitled “State of Global Air 2019: A Special Report on Global Exposure to Air Pollution and Its Disease Burden”, whose authors were The Health Effects Institute (HEI) and The Institute for Health Metrics and Evaluation (IHME). Our search found the HEI and IHME document [4], which was not published in The Lancet.

In response to the second question, ChatGPT stated that a study published in the EHP in 2018 found that an increase in nitrogen dioxide (NO_2_) was associated with a 2.5% increase in respiratory mortality in Europe. When asked about the cited study, it replied with the title, volume, and issue of the above-mentioned journal, but we were unable to locate it. The response also included a study published in The Lancet Planetary Health in 2019, entitled “Ambient Particulate Air Pollution and Daily Mortality in 652 Cities”. We found a study in The New England Journal of Medicine with the same title [5]. We asked ChatGPT to provide the study link and it sent us to a “Page not found” message. We informed it that the link was incorrect. ChatGPT apologized and then informed us that the study had been published in the journal Environmental Research and sent us a link to an article in a Urology Journal.

The articles closest to those indicated by ChatGPT were the works published by The Lancet Commissions [6] and the Global Disease Burden, published in the Lancet in 2016 [7], which contained mortality numbers and mortality increments lower than those provided by the AI.

We believe in the potential of AI, specifically as a way to provide a bridge between the non-academic population and scientists. However, we expect its results to be accurate. The citation of recognized journals was dangerous, since it led us to believe that the answers were sound. People tend to trust information providers such as big tech. We urge the ChatGPT developers to review how they feed information into the AI model and to do so sparingly. But how can this be achieved? Citing high-impact journals is a start. The next step may be teaching the AI to differentiate between an article’s introduction and its results. Whatever happens, we have to pay more attention to AI and work together to keep AI and its providers in check, in order to ensure the information they provide is accurate.
